# Experiences of Public Doctors on Managing Work Difficulties and Maintaining Professional Enthusiasm in Acute General Hospitals: A Qualitative Study

**DOI:** 10.3389/fpubh.2018.00019

**Published:** 2018-03-02

**Authors:** Andrew Leung Luk, Adrian Fai To Yau

**Affiliations:** ^1^Nethersole Institute of Continuing Holistic Health Education, Alice Ho Miu Ling Nethersole Charity Foundation, Tai Po, Hong Kong

**Keywords:** work difficulties, professional enthusiasm, doctor burnout, doctor resilence, qualitative research

## Abstract

**Background:**

Overseas studies suggest that 10–20% of doctors are depressed, 30–45% have burnout, and many report dissatisfaction with work-life balance. A local study on public doctors showed that 31.4% of the respondents satisfied the criteria for high burnout. Young, but moderately experienced doctors who need to work shifts appeared most vulnerable. This study aims to explore the experiences of those public doctors who have managed their work difficulties and maintained professional enthusiasm for references in medical education and continuing professional training.

**Method:**

Ten public doctors with reputation were invited respectively from three acute general hospitals for an in-depth interview. Interviews were audio recorded and transcribed. Content analysis was carried out to identify major themes in relation to the research questions.

**Results:**

Three themes emerging from difficulties encountered were (1) managing people, mostly are patients, followed by colleagues and then patients’ relatives; (2) constraints at work, include time and resources; and (3) managing self with decision-making within a short time. Three themes generating from managing work difficulties included (1) self-adjustment with practicing problem solving and learning good communication appeared more frequently, followed by maintaining a professional attitude and accumulating clinical experiences; (2) seeking help from others; and (3) organizational support is also a theme though it is the least mentioned. Four themes emerging from maintaining work enthusiasm were (1) personal conviction and discipline: believing that they are helping the needy, having the sense of vocation and support from religion; disciplining oneself by continuing education, maintaining harmonious family relationship and volunteer work. (2) Challenging work: different challenging natures of their job. (3) Positive feedback from patients: positive encounters with patients keep a connectedness with their clients. (4) Organization support: working with good colleagues and opportunity for continuous training.

**Conclusion:**

Some implications for medical education include, developing good communication skill for medical students and junior doctors, preparing senior doctors to be mentors, and exploring the motivating force of spirituality/religion.

## Introduction

Hong Kong is a metropolitan city with more than seven million people living in a rather small area. The healthcare services are provided by both public and private sectors. The doctor to patient ratio is 1.9 doctors per thousand population, which is lower in comparison to the advanced countries such as 3.7 in Britain, 3.3 in United States, 2.3 in Japan, 2.6 in Korea, and 3.0 in Singapore (1). There are two universities for the training of doctors. All locally trained doctors will be recruited by the Hospital Authority (HA) to be placed in the public hospitals after their graduation for residency. They mostly work in the public hospitals to gain clinical experiences and develop specialty expertise before changing to private practices. The HA is a body incorporated in the Hong Kong Special Administrative Region which manages and develops the public hospital system and offers more than 90% of all hospital services. There are 42 public hospitals and institutions scattered over seven clusters in Hong Kong. The HA employs 75,000 staff in which 6,000 are doctors (2).

Healthcare industry is one of the high risk entities worldwide because of its high volume of users, complexity of procedures, and involvement of many stakeholders. Overseas studies showed that healthcare professionals are under great stress and prone to burnout though they are providing incredibly meaningful and personally fulfilling jobs (3, 4). In a national survey of burnout in a large sample of U.S. physicians and a probability-based sample of the general U.S. population for comparison, it was found that burnout is more common among doctors than among other U.S. workers. Furthermore, doctors at frontline of care seem to be at greatest risk (5). In Hong Kong, doctors are also under great risk when performing their duties in public hospitals, where patient beds are usually fully occupied and the workload is high, particularly if they are unprepared to face crisis at work. In a cross-sectional survey of 226 valid questionnaires returned from 1,000 randomly selected public doctors in Hong Kong using the Maslach Burnout Inventory (MBI), 31.4% of the respondents suffered from high burnout. Young, but moderately experienced doctors who need to work in shifts appeared most vulnerable (6). If doctors are under great stress and prone to burnout, it not only affects their well-being, but also can lead to human errors which may cause harm to patients.

What work difficulties the public doctors are facing in Hong Kong? How do they manage these difficulties? And how they maintain their professional enthusiasm especially for those public doctors working in acute general hospitals with heavy workload? Professional enthusiasm in this study is referred to healthcare professionals, such as doctors who manage to remain consistently engaged in their career and motivate themselves for best performances. Since there has been a paucity of research in studying the resilience of public doctors in facing the high expectation of the public in the healthcare system, this study was aimed to explore the experiences of those public doctors who have managed their work difficulties and maintained professional enthusiasm for reference in medical education and continuing professional training.

## Literature Review

The well-being of doctors affected by stress and burnout has been an emerging concern in western countries for many years (7, 8). In current measures of burnout, the MBI is frequently used. Though there are arguments that MBI is limited by arbitrary cutoffs and may not have sufficient theoretical grounds (9, 10), it can be used for screening of symptoms of burnout and raise one’s awareness about the early signs (11). Overseas studies showed that doctor’s suicide rates are substantially higher compared with the general population (12, 13). However, doctors are slow to seek help (14), owing to fear of judgment, stigma, and punitive action (15, 16). Fear of stigmatization is not only present when they are performing duties as doctors, but as early as they are trained as medical students. At medical school, the competitiveness, the quest for perfection, and the fear of showing vulnerability have been cited as triggers for mental ill health. A study by Schwenk, Davis, and Wimsatt (17), on depression, stigma, and suicidal ideation in medical students in a university in U.S. revealed that 53% of respondents who reported high level of depressive symptoms were worried that revealing their illness would be risky for their career. And 62% expressed that asking for help would be admitting their coping skills which were inadequate. It is commented in this research that the characteristics of medical education emphasizing professional competence and outstanding performance may reinforce stigmatization and hinder medical students with mental problems to seek help. Furthermore, professional demand on making life and death decisions, which should not be wrong, adds extra pressure to them. For those who seek perfection, any sense of making mistakes makes them feel very anxious.

Western studies suggested that 10–20% of doctors were depressed, 30–45% have burnout, and many reported dissatisfaction with work-life balance (5, 18). Other studies showed that there were increased rates of self-medication, alcoholism, and other harmful behaviors among doctors who tried to cope with stress and burnout on their own for fear of losing their medical licenses if they reported mental stress (19–21). Though there are cultural differences, studies in Asian countries also show that doctors are under stress and prone to burnout in public hospitals. A survey study in Mainland China on 1,202 registered hospital doctors indicated that occupational stress was strongly related to burnout among hospital doctors in China. Some factors relating to burnout identified were dissatisfaction with doctor–patient relationship, working more than 40 h per week, low level of supervisor support, low coworker support, high job demands, and low reward (22). A nationwide questionnaire survey on stress, personal characteristics, and burnout among 555 first postgraduate year residents in Taiwan showed that working environment, emotional pressure, and demands from patients were their primary sources of stress. Burnout was positively correlated with stress, neuroticism, negative affectivity, disengagement coping, and weekly work hours. It was suggested that resident’s personal characteristics were closely related to stress and burnout. Therefore, in addition to assisting their work-related stress, exploring their personal characteristics should be taken into account for early identification of residents at risk of burnout (23). A cross-sectional survey on 1,000 doctors randomly sampled from the Hong Kong Public Doctors’ Association registry in which a total of 226 questionnaires returned were analyzed, 31.4% of the respondents satisfied the criteria for high burnout. Young, but moderately experienced doctors who needed to work in shifts appeared most vulnerable (6). All these studies showed that medical students, residents, and young doctors are more prone to stress and burnout with risk of depression and suicide.

In the universities, student counseling, support groups, and lectures focusing on stress reduction and coping strategies have been provided. Some medical schools in the west initiated health-promotion programs which have been reported to have positive results in reducing the negative effects of stress upon the health and academic performance of medical students (24, 25). A life skill program developed in Duke University (26) has been found to reduce hostility, cynicism, aggression, and maladaptive clinical decision-making in the U.S. medical students (27). A pilot comparison study of Chinese medical students in one university in Mainland China showed that the program was also effective (28). Studies pointed out that residents are facing lots of stressors, such as long work hours, sleep deprivation, and decline in personal and professional relationships (29–31). Resident wellness programs employing lectures, workshops, or exercises to actively educate residents about the pitfalls of burnout and the habits of wellness have been provided in some hospitals (32–34). Evidences showed that residents involved in these types of programs have demonstrated reduction in burnout scores (35, 36). Regarding strategy to help individual doctor improve their well-being, a computer-based, interactive, and individualized intervention program was found to be effective to promote behavioral change (37). The study involved 1,150 U.S. surgeons. It was a three-step interactive, electronic intervention. First, the surgeons subjectively assessed their well-being relative to other colleagues. Second, they completed the seven-item Mayo Clinic Physician Well-Being Index and received an objective, individualized feedback about their well-being relative to national physician norms. Third, they evaluated the usefulness of the feedback and whether they intended to make specific changes as a result. Relating to the organization level, provision of counseling services were necessary, it was also suggested that senior doctors should improve their supervisory skill to provide professional guidance to junior doctors; certainly role models were of paramount importance (38). Can we learn from the experiences of doctors of good reputation on how they manage their difficulties at work and maintain their professional enthusiasm?

## Materials and Methods

A qualitative case study approach was adopted. With the help of the nursing administration department of three acute general hospitals of the New Territories East Cluster in Hong Kong, three doctors who were acknowledged to be devoted in their profession were recommended for the study from their department heads between September 2014 and May 2015 from each hospital, respectively. These participants have good performances in their department and frequent compliments were received from patients and relatives. After the recommendation, research assistant invited them for an in-depth interview individually *via* email and phone call. As purposive samples in the qualitative studies, collection of data can be completed once the data approach saturation. Originally, it was planned to collect data from nine participants, in order to enrich the content of the study, one more participant was invited for interview to reach the data saturation [(39) p.87]. Totally, 10 doctors were successfully interviewed. Each interview lasted between 45 min to one and half hours.

A semi-structured interview guide (Presentation 1 in Supplementary Material) was used to facilitate the in-depth interviews, which were conducted by the first author. The topics included: the perceptions of their work, overcoming work difficulties, maintaining professional enthusiasm, impressive experiences, concept, and practice of holistic care. The full report of the research was published in Chinese (40), only the part related to their managing work difficulties and maintaining professional enthusiasm were reported in this paper. The implications for medical education and continuing professional training were discussed.

## Ethical Considerations

The study was approved by the Joint Chinese University of Hong Kong-New Territories East Cluster Clinical Research Ethics Committee (CREC Ref. no: 2014.385). All participants were given explanation of the purpose of the study. Voluntary participation and confidentiality were assured. Informed written consent was obtained from each participant including audio recording of the interview and publication of the research findings. All the records would be destroyed after the publication of the research report.

## Data Analysis

The interviews were tape recorded and transcribed. The coding approach suggested by Miles and Huberman (41), to create a general accounting scheme for codes was used. The data were then analyzed using content analysis approach (42). The majority views of the participants were grouped into categories. Consensus was obtained by the first and the second author during the process of developing the codes and categories in the data analysis stage. Finally, the results were shared with the participants for checking to ensure validity.

## Findings

### Background Information

Ten participants were interviewed, including Consultants, Associate Consultants, and frontline Medical Officers. As the Department of Medicine is the main department in the hospital, half of the participants were from this department. Others were from surgical, anesthesiological, and pediatric departments. Working experience ranged from less than 10 to 30 years; over half of them were male. Age ranged from 30 to 60. Most of them were married and had contact with a religion. Please see Table 1 for the detail distribution.

**Table 1:** Background information (*N* = 10).

Occupation	Healthcare	
Rank	Consultant	4
	Associate consultant	4
	Medical officer	2

Specialty	Medical/geriatric	4/1
	Surgical/orthopedics	1/1
	Anesthesia	1
	Pediatrics	2

Work experience	<10 years	2
	10–20 years	3
	21–30 years	5

Gender	Male	6
	Female	4

Age	30–40	3
	41–50	3
	51–60	4

Marital status	Married	9
	Single	1

Religion	Christianity	6
	Catholic	2
	Nil	2

### Difficulties Encountered at Work

The difficulties they encountered were mainly from (1) managing people, mostly of patients, followed by that of colleagues and relatives of patients. (2) Constraints at work, including time and other resources. (3) Managing self, for the need to make quick decision. Details can be seen in Table 2 and some of the descriptions were as follows.

**Table 2:** Codes, categories, and themes of difficulties encountered at work.

Themes	Categories	Codes
Managing people	Managing patients	Unrealistic expectation x 2
		Anxiety/emotion x 2
		Lack of continuous service x 1
		Non-compliances x 1
		Incurable disease x 1
		Patient’s death x 1

	Managing colleagues	Conflicts with other teams x 2

	Managing patients’ relatives	Demanding relatives x 1

Constraints at work	Limited time	Multi-tasks with limited time x 2
		Heavy workload with time limited x 2
		Limited time in out patient unit x 1

	Limited resources	Long waiting time for diagnostic procedure x 1
		Referral for other treatment takes long queue x 1
		Limited consultation rooms x 1

Managing self	Quick decision-making	Short time for critical judgment x 1

#### Managing People

##### Unrealistic Expectation

“Pretty hard, I can’t fulfill his need and truly can’t give the treatment he wants. In fact, he just wants a kidney transplant from the very beginning and requests nothing else.” (A renal associate consultant)

##### Lack of Continuous Service

“In the government or public organizations, I think it is difficult to establish rapport because you do not know the patients well. At one time the patient sees doctor A, next time sees B, then C, from A to Z; each of them would not familiarize with the patient. In this setting, it is easy to have conflict, or to increase the chance of creating conflict; everyone has their own expectations.” (A pediatric associate consultant)

##### Conflict with Other Team

“Our focuses are not the same. They just focus on operation, operation and operation. They may not have the thought that the patient is very old with many illnesses. Can one stand the operation? How much can one benefit from the operation? This kind of conflict can happen. The surgeon may have already settled with the patient stating that one must have the operation. But when we anesthetists come to see the patient, we may think that, if you should receive the operation, you may not die from the operation, but you may die from the anesthesia. Will the patient still want it? We have this kind of conflict all the time.” (An anesthesia associate consultant)

“If I really don’t agree with the specialist, usually the treatment I refer to could have really serious consequences, maybe life and death. I think I will talk with the specialist, telling him that this is not the best way. Such kind of discussion is difficult, sometimes it will end up with quarrel.” (An ICU medical officer)

##### Handling Patient’s Relative

“Some families request seeing us every day. We would repeat the same conversation every day just like replaying a recorded tape. Some keep asking questions every day. Some patients’ relatives demand you to turn the patient hourly or to help them do this or that, treating us like their domestic workers.” (A medical consultant)

#### Constraints at Work

##### Time Constraint

“There are many constraints in reality: in the setting and in the time distribution. If spend more time on one patient, that means you have to spend less time for the next one. How can we justify which patient should have a longer time, which patient’s time could be sacrificed. You will have this concern. For example, a doctor starts a ward round in the morning. After this, he has to conduct an operation, or needs to go to the clinic. If the doctor hasn’t finished the ward round on time but the patient has already been put on the operation table, this is not okay. So you have to manage the time well. Time is a constraint.” (An orthopedic associate consultant)

#### Managing Self

##### Making Decision in a Short Period of Time

“For ICU cases, one needs to make decisions in a very short period of time: intubate or not? Prescribe what kinds of drug? If you make the wrong decision, there is no return. Time is so short; this is really hard.” (An ICU medical officer)

### Managing Work Difficulties

Managing work difficulties could be categorized into three themes. (1) Self-adjustment. (2) Seeking other’s help. More people mentioned self-adjustment, among which practicing problem solving, learning good communication, and maintaining professional attitude appeared more frequently, followed by increasing clinical experiences. Other’s help came mainly from colleagues. (3) One participant mentioned that good organization support was also one of the helping resources. Details can be seen in Table 3 and some descriptions are as follows.

**Table 3:** Codes, categories, and themes of managing work difficulties.

Themes	Categories	Codes
Self-adjustment	Practicing problem solving	Solving problem x 1
		Setting priority x 1
		Faster consultation x 1

	Learning good communication	Good verbal and non-verbal communication x 2
		Selective communication x 1

	Maintaining professional attitude	Empathy x 1
		Conveying an attitude of seeking the best for patient x 1
		Respecting patient’s autonomy x 1

	Others	Accumulating clinical experiences x 2
		Reading books x 1
		Learning from patients x 1

Seeking help from others	Colleagues	Support from colleagues x 3
	Senior	Advice from senior x 1
	Patients	Support from other patients x 1
	Relatives	Support from patients’ relative x 1

Organizational support	Departmental policy	Policy to support junior x 1

#### Self-Adjustment

##### Solving Problem

“Frankly speaking, we only tackle the problem we encounter one by one.” (An anesthesia associate consultant)

##### Setting Priority

“Regarding the use of time, you have to set priority. First settle what concerns the patient most. And for matters that need to be addressed early, address them first. For some patients who need a conversation that may be more leisurely in nature or require more elaboration, or when more time needs to be devoted, I may schedule an appointment to deal with it and may even need to meet the relatives of patient as well. In general, most of the time, I, as well as my colleagues, will see the relatives after the outpatient consultation in the afternoon. We may leave late sometimes.” (An orthopedic associate consultant)

##### Good Verbal and Non-verbal Communication

“Even if you can’t cure the patient, you can maintain good communication by offering explanations to them. You know you are not able to help them; you still give them clear explanations. Your eye contact, attitude, gentle voice, and particularly, your command of medical knowledge, these can already give them a lot of support.” (A pediatric oncology medical officer)

“Should get to the point, the key point. Should know how to adjust according to the situation and how to ask appropriate questions.” (A pediatric oncology medical officer)

##### Selective Communication

“Acute cases are really difficult. The best you can do is, on their first day admission, you tell them what their conditions are like at that moment. When they are discharged, you call their relatives again and tell them what then their conditions are. In the long run, we can help them arrange diagnostic tests and follow-up management or their prescriptions have been changed. Those are all we can do ….” (A renal associate consultant)

“You can only do these on a limited and selective manner. For complicated or urgently admitted critically ill cases, you would communicate more with their families….” (A renal associate consultant)

“Besides, you will spend more time on young patients being admitted and diagnosed to have seriously illnesses. You should pay more efforts in treating them.” (A renal associate consultant)

##### Empathy

“My experience is that, if you argue with them, it doesn’t work. After being scolded by them for some time, you definitely will know that they are not pinpointing at you. They are only angry with themselves. You should keep calm, then you will have the chance of getting back the dominating role and leading them to speak slower. They are really miserable, not pretending at all. I think we ourselves or our family members may have the same experience. No matter how rich, how powerful, how senior you are, when you are sick, you are totally nothing.” (A medical consultant)

##### Demonstrate an Attitude of Seeking the Best for Patient

“All in all, make them feel that we have already tried our best to help. Many of them have a lot of resentment when they come back. As you have seen them for such a long time, they know your intention is for their benefit, they will treat you well in return.” (A renal associate consultant)

##### Respecting Patient’s Autonomy

“It’s nothing, you can ventilate. We all know these difficult cases. We tell them that they will be in trouble and need to be admitted again later for sure, putting all of us in a difficult situation. We cannot monitor them too closely. I could not really treat them like my relatives. I think this is no big deal. We can arrange a close follow-up appointment and try to pay more effort to persuade them next time. I tell my colleagues, ‘this time he/she has this kind of problem, next time see if you can think of another way to solve it.’” (A renal associate consultant)

##### Accumulating Clinical Experience

“While you sit there, you already sense something wrong about this patient; you know this is a difficult one. I really can predict from their facial appearances like a fortune teller. When he/she comes in, you already know that this one is troublesome, you will need to use more time on the consultation, you will ask more details.” (A renal associate consultant)

“I read a lot of books. And I have many years’ experience. Patients just say a few words, then I can tell what they want. I think all these count on experience.” (A pediatric oncology medical officer)

#### Seeking Help from Others

##### Support from Colleagues

“When the conditions of the case becomes more complex, referral may be needed. We may informally seek help from colleagues of the same department, see if they may have a look and give me some advice for follow-up. We may also need to call and consult other specialties such as the specialty of medicine for help, as sometimes our knowledge of that field has already been outdated, or our competence has not reached the expected level yet. There are also instances when our patients have emotional needs but we cannot afford that much time to address them. At that time, we need to ask for help too, by referring to the clinical psychologist or even chaplaincy services, for example.” (An orthopedic associate consultant)

##### Advice from Senior

“I rely on the medical knowledge I have acquired. I must know when to seek help from my seniors at appropriate times. Do not wait until the point of no return. Ask for their opinions even if you have to forget your pride. If you do not know, ask people to help. Do not just to try to save your face. But do not make yourself look like a fool by frequently asking for others’ help. Be knowledgeable. Learn how to put down your pride. And accumulate more experience.” (An ICU medical officer)

##### Support from Patients and Patients’ Relatives

“Will call their family for help, see if there is anything the family can do. Nurses will keep on persuading the patient too. And our hospital has a patient mutual help group, the Kidney Bean, for patients’ mutual contact and support. We may also call chaplains and medical social workers (MSW). As sometimes we may not afford too much time figuring out the family background of patient, MSW may take the role. They will fully grasp the patient’s background and know if there is any difficulty encountered in the family or if there is any financial issues involved, for example. All of these help.” (A renal associate consultant)

#### Organizational Support

##### Policy to Support Junior

“So we have to be executive-led. Senior staff, that is those with more working experiences are required to wait until the junior staff, MO finished their consultation. This is called executive-led.” (A medical consultant)

### Maintaining Professional Enthusiasm

From the analysis of data, four themes could be generated. (1) Personal conviction and discipline: participants were motivated by one’s belief that they are helping the needy, having the sense of vocation, and support from religion. Another category included disciplining oneself by continuing education, maintaining harmonious family relationship and volunteer work. (2) Challenging work: devoting to their jobs was also influenced by different challenging natures of their job. (3) Positive feedback from patients: positive encounters with patients keep a connectedness with their clients. (4) Organization support: good management is also a theme though it is the least mentioned. Details of the categories and codes can be seen in Table 4 and some descriptions are as follows.

**Table 4:** Codes, categories, and themes relating to maintaining professional enthusiasm.

Themes	Categories	Codes
Personal conviction and discipline	Personal conviction	Helping people in need x 3
		Sense of vocation x 1
		Response to God’s calling x 1
		Help from religion x 1

	Self-discipline	Keeping on reading x 1
		Maintaining a harmonious family x 1
		Doing some volunteer work x 1

Challenging work	Different challenging nature of work	Challenging job x 1
		Complicating job x 1
		Interesting job x 1
		Highly effective job x 1

Patients’ positive feedback	Positive encounters with patients	Patient’s appreciation x 1
		Patient’s trust x 1
		Patient’s connectedness x 1

Organization support	Good management	Good leader x 1
		Good colleagues x 1
		Recognition and promotion x 1
		Official release for training x 1

#### Personal Conviction and Discipline

##### Helping People in Need

“If I go into private practice, I need to have a business mind, needn’t I? Working in private practice has its own difficulties. I can see that you can only serve those who can afford because you need to pay for the rent, employees and many other things. If I serve in public hospitals, this is a good thing, you can really help those in need of your expertise.” (A medical consultant)

“I think both those who stay in or leave the public hospitals have enthusiasm to serve. The question is they need to decide what kinds of patients they wish to serve. I think everyone has their own position and can decide what types of patients they serve. In public hospitals, you can actually help those with less social resources, that is, those who cannot afford much to see doctors. So you are really helping the lower social class.” (An orthopedic associate consultant)

##### Sense of Vocation

“Why my colleagues become a doctor? There are many different reasons. Some have good academic achievement but feel that there are little choices. For me, this is an honor, a special social status…If you feel that this is a vocation, you would have a sense of mission and exhibit special behaviors. If you feel that you are just a paid employee, you would have a poor attitude and not ready to walk an extra mile. If there should be 150 cases divided among 5 doctors, each one should see 30. You would not think that this talent of healing carries responsibilities too.”

“I always think that it is not easy to become a doctor. It depends on a lot of blessings to enable us to become one. If you are fortunate, you become a doctor. If you are not, you become a patient. I always ask myself why I can be a doctor standing at the end of the hospital bed treating patients while others can become patients lying at the other end. If there should be a turn in fate, you can become a patient staying in this same hospital like I did. If you should become a patient you can feel the helplessness. You are so blessed by having good health, knowledge and logic to help others. If you do not know how to treasure these blessings, you would not have the heart to serve others. I think this attitude is quite important.” (A surgical consultant)

##### Help from Religion

“I need my religious faith to support and refresh me…. This is an important motivator…. Here is like a castle, a medieval castle with a wall; I am the gate keeping soldier, trying my best to guard it. Why I use this metaphor? Because there is a verse in the Bible: “Unless the Lord watches over the city, the watchmen stand guard in vain” (Psalm 127:1). I know I am the guard trying my best; the Lord working with me is the most crucial point. If He is beside me, I only need to stay alert. I have always been like this up to today, always trying my best. If I do not think this way, I would feel very tired and simply cannot continue. I would continue personal devotion and praying everyday in order to refresh myself. I understand my role. My present level of knowledge is not perfect. I would endeavor to aim for the best, but how the patient would respond is out of my control. I surely know that when we do the best, the rest is up to God. I know my limitations. But I would not stand back and let Him fix things because of this thought. We have to be clear about this.” (An ICU medical officer)

#### Challenging Work

##### Interesting Job

“I think I find my job interesting and it can help my patients to some extent. I find it interesting because I can see some rare cases. I notice that there are a lot of research being done in various parts of the world. Then you can get to know their pathological bases; perhaps there will be new treatments available. I then can have chances to see how new development benefit my patients. Perhaps you can diagnose a particular condition and give the most appropriate treatment. You can improve the management of their diseases; this is my greatest motivation.” (A pediatric associate consultant)

“If you can achieve this, it would give you the greatest satisfaction. Even if you cannot cure some diseases you can still give them another kind of support, namely, care. You may help the family to look after the sick child. Even if you cannot cure them, you can still actively listen to them. Let them feel that they are being cared for. At times I feel that there are other possible and practical things you can do.” (A pediatric associate consultant)

#### Patients’ Positive Feedback

##### Appreciation from Patients

“A simple word of thanks, or a word of appreciation like: ‘Ah, you have spent a lot of time on me today.’ The patients feel happy themselves. Or they may say: ‘doctor, I come to see you today.’ They indicate they want to see you because you would listen to them. They may not praise you directly. They may send you a card or a cake for the whole team at Christmas. These are very encouraging. These gifts, though small, can give you positive feeling.” (An anesthesia associate consultant)

##### Connection with Patients

“I had attended patients during my pregnancy period. After my child delivery I returned to work, they would congratulate me. This indicated that rapport had been developed between me and the patients. Somehow, after several months to half a year, when they returned to see you again, they would recognize your changes. Then they would tell you their changes too. I feel that this would motivate you to continue to listen to patients with a heart.” (An anesthesia associate consultant)

#### Organization Support

##### Good Leader

“If you have a good boss, you will be motivated to perform better as you believe your boss still outperforms you that much. You simply have no reason accepting that you, as a subordinate, lag behind your boss that far. That is, your boss should be a good role model, a very knowledgeable and good person. When you still see the distance between your boss and you, you will want to…You don’t want to disgrace your boss. Or put it in another way, it gives you the motivation to move forward. Your boss should let the subordinates know he or she trusts them and they have already done a good job. He or she maintains a hands-off way of management.” (A renal associate consultant)

##### Official Release for Training

“To have official release for study, like attending some courses, I think it is needed. In fact, what if the management can be more far-sighted?…On one hand, the staff can take a rest. On the other hand, he or she can bring in more new knowledge, information and viewpoints.” (An ICU medical officer)

## Discussion

### Difficulties Encountered at Work and Ways for Managing

What work difficulties the public doctors are facing in Hong Kong? The findings of this study showed that the main difficulty is from patients with time constraint. With different expectations from patients, plus the heavy workload, public doctors face tremendous stress. When one is sick, be it due to physical or mental problem, it will lead to bodily disequilibrium with emotional instability. The condition will even be worse when the persons have to be admitted to hospital as patients for treatment. The patient’s relatives are also anxious for their loved ones, particularly for the dying patient where hospital will be the place for ending life. Public doctors will see people from all walks of life with different kinds of diseases and various expectations. It is understandable that caring for them would be difficult in acute public hospitals where the workload is usually heavy. They have to fulfill their roles in diagnosis and treatment within certain time limit. The stress is even higher in times of medical emergencies for saving life. The findings in this study illustrated why public doctors find it difficult to take care of patients’ needs, especially for young doctors who have not gained sufficient clinical experiences. As echoed by Sung (43), Vice Chancellor, the Chinese University of Hong Kong, the turnover rate of beds, burdensome, and time-consuming paperwork, the fear of malpractice litigation, may make the younger generations of doctors feel more difficult to find satisfaction and self-actualization in the clinical job and become physically and emotionally exhausted. It is consistent with an overseas study that more than 1/4 of young doctors experience burnout (20). A local cross-sectional survey of public doctors also showed that 31.4% of the respondents satisfied the criteria for high burnout. Young, but moderately experienced doctors who need to work in shifts appeared most vulnerable (6).

Regarding managing difficulties at work, this study revealed that practicing problem solving in setting priorities, learning good communication with patients, maintaining professional attitude, and accumulating clinical experiences are strategies used by them in self-adjustment. While seeking help from colleagues and organization support are also important. The findings in this study are consistent with some of the findings of the study by Jensen et al. (44) about building physician resilience. Self-adjustment starts from acceptance of professional demands and awareness of personal limitation both in time and expertise. With balance and prioritization, one can set limits and manage the workload. Seeking professional support from peers, consultants, and multidisciplinary teams together with personal support from family members and friends are also highlighted. However, good communication skill is very much prominent in this study, since good communication can prevent misunderstanding. Furthermore, an empathetic attitude can build up trust, lessen complaints, and provide better care instead. The importance of communication with patients are frequently mentioned during medical education, however, it is usually more focused on gathering accurate data in facilitating diagnosis. Therapeutic communication with a caring attitude is an important value which needs to be developed, practiced, and become a habit of showing empathy in doctor–patient relationship. As suggested by Sung (43), it is difficult to teach values and ethics in classrooms, it has to be taught or caught by examples. Setting up models by senior doctors can support junior doctors in their initial career path and prepare them to be caring seniors in the future.

The finding of this study also showed that accumulating clinical experiences will help to manage the time constraints for medical consultation. It is understandable that practices make perfect. Doctors learn to recognize different patterns of diseases’ presentations, so with more experiences they can make the diagnosis faster and more accurately. However, junior doctors need more time to practice, consolidate their professional knowledge and skills. Generally speaking, in a specialist medical clinic, one doctor has to see around 30 patients at least within 3 h in an afternoon session, spending average 6 min for each patient. Under this time constraint, it is difficult for them to have better understanding and therapeutic communication with patients. Adding more resources to support junior doctors remains to be an important issue to be solved by the HA.

### Maintaining Work Enthusiasm

With regards to maintaining work enthusiasm, the findings in this study showed that it comes mainly from personal conviction in the belief of helping people in need and a sense of vocation; self-discipline in terms of continuing education, keeping a harmonious family, and participating in some voluntary works. All these are consistent with findings and suggestions made by both local and overseas studies (44–46). Most of the participants in this study are with religious background; getting help from religion may become one of the elements in keeping their work enthusiasm. Besides, many studies support religion as a protective factor in individual support and coping (47–50). Second, having a challenging job is also a motivator.

This study also revealed that positive feedback from patients is one motivator for keeping the working spirit. It is consistent with findings from some local studies that patients’ recovery and their appreciation will definitely encourage healthcare professionals to stay serving in the public hospitals and continually committing in their helping professions (40, 51, 52). Regarding another finding of the support from organization, the HA of Hong Kong has set up a psychological care department called “Oasis” to offer psychological support to staff by means of conducting courses for personal growth and managing stress. Furthermore, it also provides individual counseling and crisis intervention after clinical incidents in hospitals. However, due to the heavy workload in acute general hospital, it is not easy for department heads to officially release staff to attend these courses. Using a computer-based, interactive, and individualized intervention for promoting well-being, such as the one conducted by Shanafelt et al. (37) for the U.S. surgeons, can be considered as an alternative method to be implemented for professional staff locally. They can join the training according to their appropriate time.

## Conclusion

There are some implications from this study for medical education and continuing professional training. First, developing good communication skill in terms of conveying a caring attitude is important in medical education both for medical students and young doctors. Second, preparing senior doctors to become good leaders and mentors are necessary in helping young doctors to adjust faster in the beginning of the career life. Finally, the motivating force of religion in enriching one’s life is worth further exploration.

## Ethics Statement

The study was approved by the Joint Chinese University of Hong Kong-New Territories East Cluster Clinical Research Ethics Committee (CREC Ref. no: 2014.385). All participants were given explanation of the purpose of the study. Voluntary participation and confidentiality were assured. Written consent in accordance with the Declaration of Helsinki was obtained from each participant. All the records would be destroyed after the publication of the research report.

## Author Contributions

AL is responsible for the study design, data collection, data analysis, and report writing. AY takes part in study design and data analysis.

## Conflict of Interest Statement

The authors declare that the research was conducted in the absence of any commercial or financial relationships that could be construed as a potential conflict of interest.
