# Supplementary material for: Experiences of Public Doctors on Managing Work Difficulties and Maintaining Professional Enthusiasm in Acute General Hospitals: A Qualitative Study
Source: Front Public Health. 2018 Mar 2;6:19. doi: 10.3389/fpubh.2018.00019 (PMC5840166; doi:10.3389/fpubh.2018.00019)
Supplement: Supplementary file 1 [file presentation_1.PDF]

Semi-interview guide for
"Best practice of holistic care: A case study approach"

I. Personal background:

Profession _______________ Specialty ______________
Working experiences _______ years Year in AHNH/NDH/PWH _____
Sex _________________ Age __________
Marital status ______________ Religion ______________

II. Semi – interview guide:

1. Can you briefly introduce yourself?

2. How long have you been working in the present unit?

3. What kind of care do you usually delivered to your clients?

4. What do your clients usually respond to your services?

5. How do you view your care delivered to your clients?

6. What difficulties are encountered in the delivery of your care?

7. How do you overcome these difficulties?

8. Can you share some impressive experiences of the care to your clients?

9. How can you maintain enthusiasm in your own work?

10. What is your view about holistic care?

11. Is there anything you would like to share about holistic care being delivered in acute hospital settings?
